# Retrospective Analysis of Cervical Cancer Treatment Outcomes: Ten Years of Experience with the Vaginal Assisted Radical Laparoscopic Hysterectomy VARLH

**DOI:** 10.1155/2022/5163886

**Published:** 2022-01-10

**Authors:** R. Wojdat, E. Malanowska

**Affiliations:** ^1^Clinic for Gynecology and Obstetrics, Mathilden Hospital Herford, Renntormauer 1-3, 32052 Herford, Germany; ^2^Department of Gynecology, Endocrinology and Gynecologic Oncology, Pomeranian Medical University, Unii Lubelskiej 1, 70-001 Szczecin, Poland

## Abstract

**Background:**

LACC trial demonstrated inferiority of laparoscopic approach for the treatment of early-stage cervical cancer. There are still limited data from retrospective trials regarding whether survival outcomes after laparoscopic radical hysterectomy are equivalent to those after open abdominal radical hysterectomy. In this study, we present results of combined vaginal radical laparoscopic hysterectomy in the treatment of early-stage cervical cancer.

**Methods:**

This retrospective study was carried out at the Department of Gynecology in Mathilden Hospital (Herford, Germany). Between January 2008 and April 2018, all the patients with invasive cervical cancer who underwent combined vaginal assisted radical laparoscopic hysterectomy (VARLH) without the use of any uterine manipulator were enrolled to the study.

**Results:**

A total number of 124 patients with diagnosis of invasive cervical cancer were enrolled in the study. All of the patients underwent minimally invasive surgery and were divided according to FIGO 2019: stage IA (25.9%), IB1 (25.0%), IB2-IIB (28.4%), and III/IV (20.7%). Overall, the mean age of the patients was 51.84 years. After a study collection, a median follow-up was 45.6 (range 23.7-76.5) months. The 3- and 5-year disease-free survival rates for early-stage cervical cancer were both 98%, and the 3- and 5-year overall survival rates were 100% and 97%, respectively. We have not observed any recurrence in our study group of patients with early-stage cervical cancer.

**Conclusions:**

Combined VARLH can be considered a safe and effective procedure for the treatment of early-stage cervical cancer. Surgical strategy with oncological principles determines the quality and long-term success of the operation in early cervical cancer regardless of laparoscopic approach.

## 1. Introduction

In a tragic way, the Laparoscopic Approach to Cervical Cancer (LACC) trial changed the development of laparoscopic surgery for early-stage cervical cancer [1]. Unfortunately, in 2018, it has also altered clinical practice significantly [2–4].

Total radical laparoscopic hysterectomy (TRLH) for early-stage cervical cancer was carried out with increasing frequency for almost three decades [5, 6]. Comparing to open surgery, laparoscopic approach was shown to have shorter operative times and hospital stays and fewer postoperative complications rates [5–7]. Therefore, several studies were conducted to explore this topic and bring back laparoscopic surgery to its rightful place [8–10].

Almost-forgotten vaginal hysterectomy has been replaced by robotic or laparoscopic techniques [10, 11]. Technical feasibility and growing experience with laparoscopic lymphadenectomy have facilitated a revival of radical vaginal hysterectomy. Thus, the Schauta-modified vaginal assisted hysterectomy has become more useful in the light of current research [12]. The procedure was associated with a decreased postoperative mortality when compared with the abdominal route that was invented by Wertheim [13–15]. However, the lack of experience in performing this technique and evidence of its oncological efficacy needs further analysis to be considered a first-choice procedure.

The change of clinical practice in early-stage cervical cancer drove us to report on our experience and assess the efficacy of combined vaginal assisted radical laparoscopic hysterectomy (VARLH) for early-stage cervical cancer.

## 2. Material and Methods

REACCT is a retrospective observational study analysing the outcomes of combined vaginal assisted radical laparoscopic hysterectomy (VARLH) in the treatment for early-stage cervical cancer. The diagnosis was made at Department of Gynecology in Mathilden Hospital Herford-Cancer Centre of Excellence (certified Centre of Cervical Dysplasia).

Our study involved all 124 of the patients with the diagnosis of cervical cancer (with initial stage I-IV according to the International Federation of Gynaecology and Obstetrics (FIGO 2019)) who underwent VARLH between January 2008 and April 2018.

After this period, we switched to open surgery for cervical cancer as a favourable technique (according to LACC Trial). We offered all patients comprehensive preoperative patient-centered counselling providing them with information as reported in the recent literature (LACC).

Patient follow-up was updated in the third and fourth quarter of 2020 using phone calls and during clinical visits.

The study was approved by the Ethics Committee on Clinical Studies of Medical University of Münster (UKM).

### 2.1. Eligibility Criteria for Surgery

Patients with early stage of cancer, stage IA1-IB1, were qualified for surgery (VALRH). Patients with stage IIA-IV (FIGO) were treated with additional personalized treatment (primary or palliative chemoradiotherapy after laparoscopic staging). All of the patients were treated by combined VARLH (lymphadenectomy with ICG sentinel mapping) with respect to disease-free survival (DFS) and overall survival (OS).

### 2.2. Surgical Technique

VARLH was performed by a senior skilled surgeon (RW). The preoperative routine placement of ureteral double-J catheters as a prophylactic of ureteral injury was performed to all the patients.

Women had their pigtail catheter removed directly in the operating room at the end of the procedure, or when the gynecologist judged that for any reason prolonged catheterization was necessary.

The SLN biopsy technique was as follows: In the beginning, the vaginal part of the surgical procedure the patient was placed in a lithotomy position and ICG (indocyanine green) was injected into the cervix with the 2-quadrant option at 3 and 9 o'clock, after closing the vaginal cuff.

**Figure 1:**
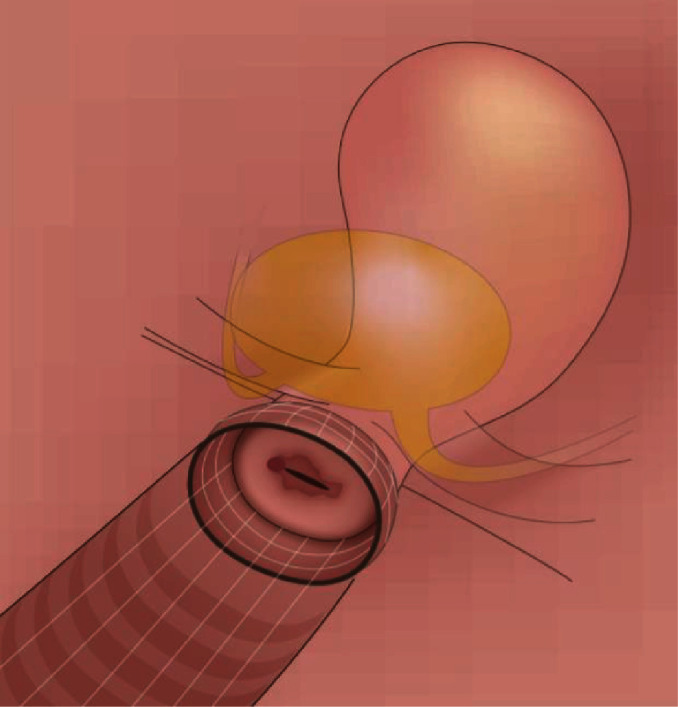
Schematic drawing of the vagina and uterus with prepared vaginal cuff. Anatomical landmarks (parametria, ureters, and urinary bladder) are depicted in this figure.

All surgical procedures preserved surgical and oncological safety with “tumor no-touch technique” (gentle surgery, without using vaginal manipulator and without injury to the uterine surface). The vaginal wall was grasped exclusively with blunt clamps. Circular, bloodless incision was made with the use of electrocautery (Figure 1).

Prophylactic antibiotics were routinely administered intravenously immediately prior surgery with a single shot dose of cefuroxime 1.5 g i.v., if there were no contraindications.

All patients received a risk-adjusted amount of low molecular weight heparin, e.g., enoxaparin 0.4 ml s.c.

**Figure 2:**
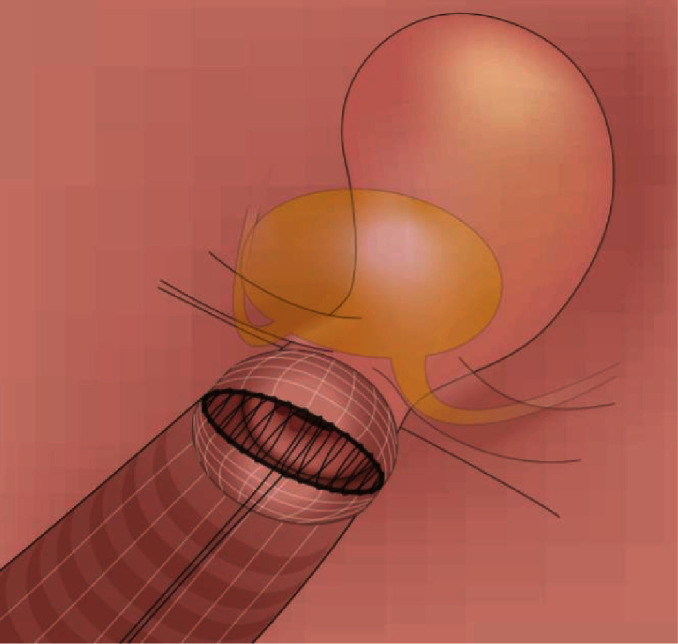
Schematic drawing of the sutures applied on the vaginal cuff. Closure direction, from the outside to the middle.

**Figure 3:**
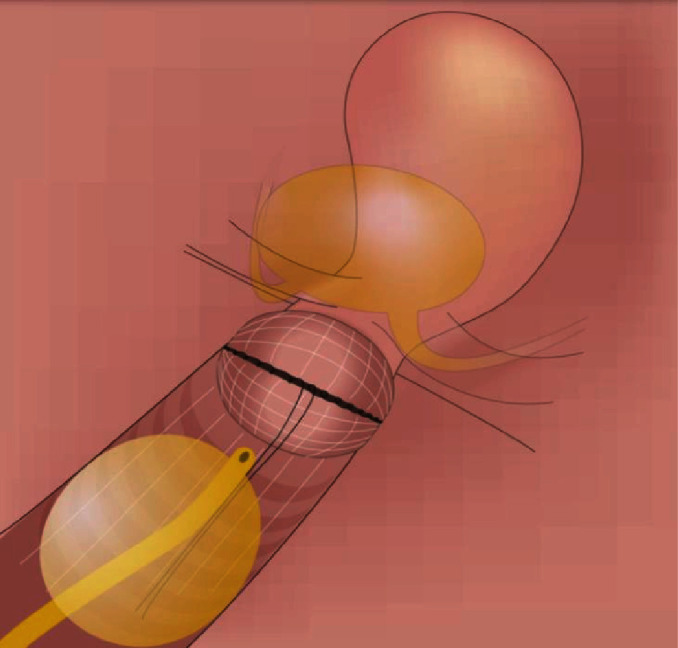
The vaginal cuff completely covers the cervix. Sutures are left in the vagina in order to remove the uterus afterwards with the pull-out technique. The vaginal canal is blocked with a urine catheter.

Selected surgical steps of VARLH were as follows:

1. Step 1 (Figure 1): circular cut of the vaginal cuff above the cervix (without the use of manipulator)

(2) Step 2 (Figure 2): covering the cervical tumor with vaginal cuff and application of continuously overturned nonabsorbable braided polyester suture Ethibond 1-0 (after mobilizing the vagina in Step 1)

(3) Step 3 (Figure 3): the avoidance of uncontrolled gas evacuation with the use of 22 Ch urine catheter (filled with 50-80 ml NaCl).

### 2.3. Statistical Analysis

Statistical analysis was performed with SPSS version 25.0 for Windows (IBM). Categorical variables are presented as frequency and percentage, while continuous variables are presented as mean and standard deviation (SD) or median (interquartile range), as appropriate. The Kaplan-Meier survival analysis was carried out to estimate mean and overall survival (OS) and disease-free survival (DFS), with the 95% confidence interval (CI), as well as to analyse factors associated with survival (logrank tests). Results are presented as mean (95% CI) survival with the logrank test. An analysis of variance (ANOVA) was used to analyse normally distributed numerical variables, while the chi-square tests were used to analyse categorical variables. The level of statistical significance was set to 0.05 to reject null hypothesis.

## 3. Results

All the patients were diagnosed with a histologically confirmed cervical cancer in the Cancer Centre of Excellence at Mathilden Hospital Herford. 124 patients fulfilled the inclusion criteria of invasive cervical cancer. The mean (SD) age was 51.84 years (SD: 15.41, median: 47.5). We lost 8 patients in the follow-up; thus, retrospective analysis included 116 patients. There was no conversion to laparotomy necessary in any patient. We did not observe any complications during the surgery, increased intraoperative blood loss, big vessels, or genitourinary tract injury. Lymphocele occurred in 2 patients in long-term postoperative period.

Tumor characteristics describes Table 1 (according to FIGO 2019 for cervical cancer) stage IA (25.9%), IB1 (25.0%), IB2-IIB (28.4%), and III/IV (20.7%). The majority of the participants were grade G2 (47.4%) or G3 (38.8%) (Table 2). Median (IQR) follow-up time was 45.6 (23.7-76.5) months.

Table 3 depicts the distribution of patients in IB1 and IB2 groups.

In one case, postoperative radiochemotherapy was necessary in the group of IB1 patients. In the IB2 FIGO group, 5 from 6 cases were indicated to postoperative radiochemotherapy.

The *DFS* for *patients* with stage IA-IB1 (45) disease was 98% after 5 years. The DFS for 25 in this group after 5 years was 98%. 18 of these patient's follow-up data of at least 5 years' duration are available. They were not included in the analysis, because we obtained data after primary registration was finished. According to our knowledge, all of the patients are in good physical condition are disease-free.

**Figure 4:**
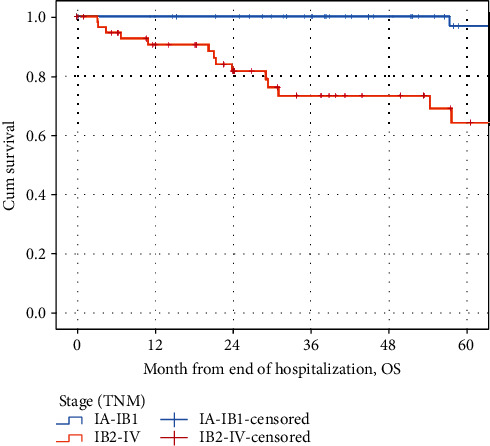
The Kaplan-Meier curves by grading for overall survival (OS) by stages 1A-1B1 and 1B2-IV.

**Figure 5:**
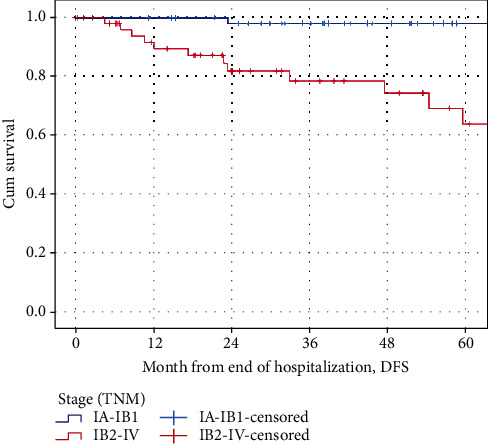
The Kaplan-Meier curves by grading for disease-free survival (DFS) by stages 1A-1B1 and 1B2-IV.

Figures 4 and 5 show the Kaplan-Meier curves by grading for overall survival (OS) and disease-free survival (DFS) by stages 1A-1B1 and 1B2-IV. Patients of stages 1A-1B1 were significantly younger than patients of 1B2-IV (45.47 vs. 58.44 years, *F* = 24.774, *p* < 0.000).

The mean (95% CI) overall survival was 150.62 months (95% CI: 144.63-156.62) for stages 1A-1B1. The mean overall survival was 104.63 months (95% CI: 83.87-125.40) for stages 1B2-IV. The mean OS decreased significantly with TNM stage (logrank test, chi^2^ = 18.285, *p* = <0.001).

The mean DFS was 151.23 (95% CI: 146.36-156.09) months for stages 1A-1B1. Thee mean DFS was 105.56 months (95% CI: 83.74-127.39) for stages 1B2-IV. The mean DFS decreased significantly with TNM stage in case of recurrence (logrank test, chi^2^ = 16.463, *p* < 0.001).

OS and DFS rates are compared (Tables 4 and 5) with the respective results of the LACC TRIAL (reference), open surgery (reference), and Koehler (reference).

The age and stage distribution of the patients in our population corresponds to normal distribution of morbidity [16].

## 4. Discussion

In 2018, at the Society of Gynecologic Oncology Annual Meeting on Women's Cancer, Ramirez et al. presented the results of the LACC Trial and thus casted a shadow on the importance of laparoscopic surgery in the treatment of early-stage cervical cancer [1].

Minimally invasive surgery many times proved its advantages and has overtaken open surgery as the choice of procedure with regard to the complication rate and period of convalescence time [5–7], especially for the treatment of cervical cancer, where it seemed to maintain the untouched position [5–8]. Established as a safety and effective procedure with relatively high overall survival rate, laparoscopic surgery has gained many advocates [8–10]. When faced with studies that contradict accepted practice, members of medical community assumed a defensive stance. After these unexpected results, they started an extensive analysis [2–4, 8]. Therefore, the question arises: what determines success in the treatment of early-stage cervical cancer? Is it really the matter of surgical access, or maybe should we take a closer look how the surgery per se impact the efficacy of treatment?

Some particular technical aspects of the MIS approach impact oncological safety and possible actions can be taken to improve the quality of surgical care. There is still an ongoing discussion regarding the use of uterus manipulator in gynecological oncology and its influence on the spread of tumor cells [9, 17]. In some studies, in patients diagnosed with endometrial cancer, uterine manipulator was associated with a worse oncological outcome [18, 19]. Also, other investigators avoid the use of a uterine manipulator during minimal invasive radical hysterectomy in the case of intraoperative tumor injuries [20, 21]. However, Nica et al. reported that the use of an intrauterine manipulator in patients with early cervical cancer was not an independent factor associated with rate of recurrence [22].

Interestingly, in a nationwide German survey, more than 50% responders answered that possible reasons and explanations for the inferior outcome of the MIS group in the LACC trial was the use of manipulator and wrong surgical technique [23]. Unfortunately, the results of NOGGO survey pointed laparotomy as a preferred surgical technique in the treatment of cervical cancer, and vaginal hysterectomy took the last place [23]. No better results were achieved by Wenzel et al.'s research group [24]. Only 33% of laparoscopic hysterectomies were performed before LACC trial came out [23]. How then surgical treatment really looked like before the pre-LACC era?

Similar controversies rose when FDA warned about the cancer-spreading risks of power morcellator devices used in gynecological surgery, which also resulted in a decrease of minimally invasive surgery [25]. All extirpating procedures used for hysterectomy, whether performed with laparotomy or laparoscopy, involve the risk of disseminating malignant cells in the abdominal cavity. However, gentle surgery, without unnecessary manipulations and without injury to the uterine surface, could significantly reduce this risk [26].

The modified Schauta procedure has been shown a high cure for stages IB to IIA cervical cancer in previous studies [27, 28]. This procedure consists of a radical hysterectomy performed vaginally without the need for a lateral perineotomy [29]. Our modification does not involve the “click maneuver” (a method that allows a vaginal exposure of the ureter). When vaginal part of the procedure was finished, visualization and preparation of both ureters were done from laparoscopic approach. Routine preoperative bilateral ureteral catheterization was helpful for intraoperative ureter identification.

Combined laparoscopic-vaginal approach offers surgical safety and allows to avoid contamination with cancer cells by covering the cervical tumor with vaginal cuff. Furthermore, with an application of continuous suture, we avoid potential dissemination of tumor cells by gas evacuation. Other techniques, like vaginal closure with the surgical stapler, were described in the literature to prevent tumor spillage [30, 31].

Our experience in laparoscopic surgery has grown over the years, and we observe a rapid advancement of medical technology. This led us to apply indocyanine green (ICG) to identify sentinel lymph nodes in oncological gynecology. SLN mapping is routinely performed in our department since 2010. Before ICG, we used the combination of blue dye and radioisotope techniques with Technetium-99. However, we did not change the surgical method, which is constant since many years. Sentinel lymph node mapping with ICG in cervical cancer followed by systemic pelvic lymphadenectomy was helpful with intraoperative decision-making process. The information about lymph node status given by the ultrastaging allowed us to carefully select a group of patients appropriate for multimodal treatment and decrease the risk of complications of unnecessary surgery [32–34].

The results of LACC trial showed lower disease-free survival (DFS) and overall survival (OS) in the minimally invasive surgery (MIS) arm [1]. The 4.5-year DFS rate was 86% for the MIS arm compared with 96.5% for the OPEN arm [1]. In our study the 3- and 5-year disease-free survival rates for early-stage cervical cancer were both 98% and the 3- and 5-year overall survival rates were 100% and 97%, respectively.

In a multicenter analysis, Köhler et al. achieved over 95.7% disease-free and 97.6% overall survival in long-term follow-up, similar to the laparotomy arm of the LACC trial and our results [35]. Data in Tables 4 and 5 depicts that the disease-free survival rate (DFS) and the overall survival (OS) rate between the studies did not differ significantly. It was 99% (3-year OS) for laparotomy arm in LACC trial, 98.5% for multicenter trial, and 100% OS for Mathilden Hospital [1, 35]. In our study, the 3 years of 100% OS was observed for IA-IB1 stage of cancer. In LSC arm of the LACC trial, this number was 93%. We also reported no recurrence at final follow-up. According to these results, we are of the opinion that combined VARLH provides a safe procedure with good clinical outcomes.

**Figure 6:**
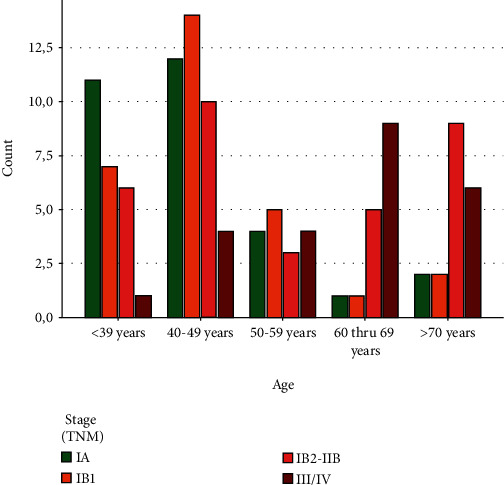
Age-dependent distribution of patients according to the stages of the disease.

Figure 6 shows the distribution of the age-dependent patients to the corresponding stages of the disease. Earlier stages can be found more frequently in younger patients, while more advanced stages are more likely to be found in older patients.

Perhaps, when evaluating our results according to old FIGO staging system, we would define incorrectly more patients in early-stage cervical cancer group. At the time, our results would be worse, which only proves that the inferior border of 2 cm according to FIGO 2019 is justifiable.

From our perspective, the implementation of the new FIGO 2019 classification enables us to make a better decision about a stage adapted therapy. In other words, it helps us better to avoid unnecessary multimodal therapy [36, 37]. Our findings are in good agreement with previous results [35]. Although the number of groups differs, the results show a clear tendency.

We expect that the new classification will be a helpful tool for better risk stratification of cervical cancer patients and that it will facilitate more personalized treatment recommendations.

Presented study is an evaluation of a single institution's experience of laparoscopic radical hysterectomy. The limits of our study are the number of patients with early-stage cervical cancer. Nevertheless, we want to point out that 124 patients with the diagnosis of invasive cervical cancer were diagnosed and treated in one center. This is quite a large sample size as compared to other studies where two or even three centers involved about 200 or less patients [23, 38]. Collecting more data regarding the efficacy of laparoscopic treatment for early-stage cervical cancer will no longer be possible due to changed clinical practice. For this reason, we need prospective randomised trials including preservation of oncological safety to analyse the topic more precisely and compare the results.

Treatment method should be selected individually, but oncological carefulness has to address the vast majority [39, 40]. Promising results of our study prove that laparoscopic surgery should not be excluded in the treatment for early-stage cervical cancer. We have to look closer for the best therapy we can offer to our patients. However, by questioning the minimally invasive surgery in the treatment of early-stage cervical cancer, we may take them the possibility, which in the end may turn out to be the best.

## 5. Conclusion

Presented combined VARLH technique should be considered a safe oncological intervention in the treatment of early-stage cervical cancer.

Surgical strategy with oncological care is a key to success in the treatment for cervical cancer.

## Figures and Tables

**Table 1:** Distribution of FIGO-stages (percent (%)).

Stage (FIGO 2019)		Frequency	Percent	Valid percent
Valid	IA	30	25.9	25.9
	IB1	29	25.0	25.0
	IB2-IIB	33	28.4	28.4
	III/IV	24	20.7	20.7
	Total	116	100.0	100.0

**Table 2:** Distribution of histological grade (percent (%)).

Grading		Frequency	Percent	Valid percent
Valid	G1	16	13.8	13.8
	G2	55	47.4	47.4
	G3	45	38.8	38.8
	Total	116	100.0	100.0

**Table 3:** Distribution of therapy in stage 1B1 and 1B2 (unimodal: patients who underwent surgical therapy only, multimodal: patients who underwent (additionally) radiochemotherapy).

Stage FIGO 2019			Ratio of therapy: single cases of unimodal/multimodal therapy
Valid	Frequency of cases	Percentage from all patients	
IB1	29	25.0	28/1
IB2	6	5.2	1/5

**Table 4:** Comparison of OS in different studies.

OS	Follow-up	3-year OS No. at risk %	5-year OS No. at risk %
LSC/robot arm in LACC trial	2.5 years	93.8%, 150 (47%)	n/a, 5 (2%)
Laparotomy arm in LACC trial	2.5 years	99%, 136 (44%)	n/a, 7 (2%)
Multicenter results Chr. Köhler et al.	>8 years (99 months)	98.5%, 306 (78%)	97.6%, 265 (68%)
MH Herford 2010-2020 R. Wojdat et al.	3.8 years (45.6 months)	IA-IB1: 100%, 45 (75%) IIB-III/IV: 74%, 22 (39%)	IA-IB1: 97%, 25 (44%) IIB-III/IV: 55%, 11 (20%)

LACC: Laparoscopic Approach to Cervical Cancer; OS; overall survival.

**Table 5:** Comparison of DFS in different studies.

DFS	Follow-up	3-year OS No. at risk %	5-year OS No. at risk %
LSC/robot arm in LACC trial	2.5 years	87.1%, 142 (47%)	n/a, 5 (2%)
Laparotomy arm in LACC trial	2.5 years	97.1%, 134 (43%)	n/a, 7 (2%)
Multicenter results Chr. Köhler et al.	>8 years (99 months)	96.8%, 306 (78%)	95.7%, 264 (68%)
MH Herford 2010-2020 R. Wojdat et al.	3.8 years (45.6 months)	IA-IB1: 98%, 45 (75%) IIB-III/IV: 79%, 22 (39%)	IA-IB1: 98%, 25 (44%) IIB-III/IV: 53%, 11 (20%)

DFS: disease-free survival; LACC: Laparoscopic Approach to Cervical Cancer.

## Data Availability

All data generated or analysed during this study are included in this published article.
